# A Biopharmaceutical Perspective on Higher-Order Structure and Thermal Stability of mRNA Vaccines

**DOI:** 10.1021/acs.molpharmaceut.2c00092

**Published:** 2022-06-17

**Authors:** Marek Kloczewiak, Jessica M. Banks, Lin Jin, Mark L. Brader

**Affiliations:** Moderna, Inc., 200 Technology Square, Cambridge, Massachusetts 02139, United States

**Keywords:** lipid nanoparticle (LNP), COVID-19, mRNA formulation, RNA delivery, circular dichroism (CD), differential scanning calorimetry (DSC)

## Abstract

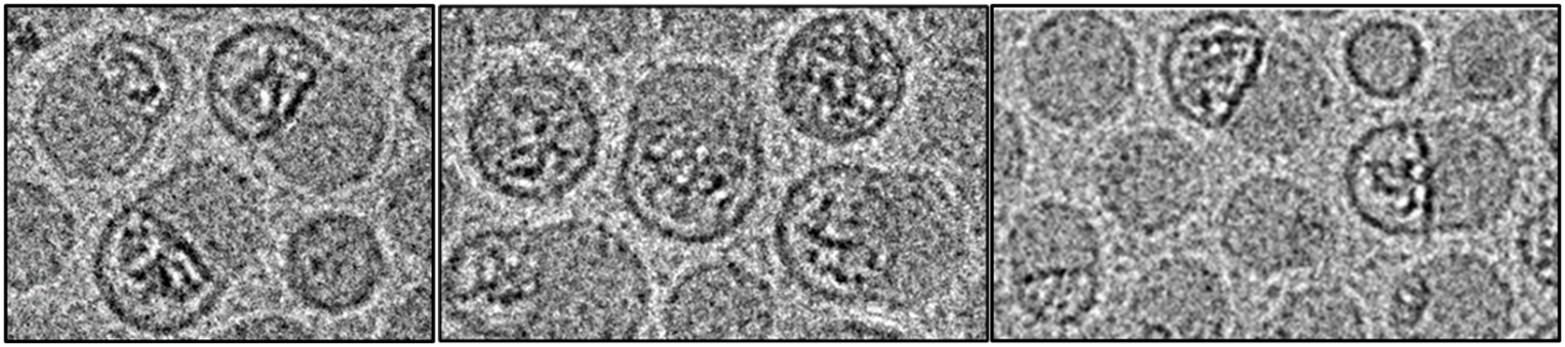

Preservation of the integrity of macromolecular higher-order structure is a tenet central to achieving biologic drug and vaccine product stability toward manufacturing, distribution, storage, handling, and administration. Given that mRNA lipid nanoparticles (mRNA-LNPs) are held together by an intricate ensemble of weak forces, there are some intriguing parallels to biologic drugs, at least at first glance. However, mRNA vaccines are not without unique formulation and stabilization challenges derived from the instability of unmodified mRNA and its limited history as a drug or vaccine. Since certain learning gained from biologic drug development may be applicable for the improvement of mRNA vaccines, we present a perspective on parallels and contrasts between the emerging role of higher-order structure pertaining to mRNA-LNPs compared to pharmaceutical proteins. In a recent publication, the location of mRNA encapsulated within lipid nanoparticles was identified, revealing new insights into the LNP structure, nanoheterogeneity, and microenvironment of the encapsulated mRNA molecules [Brader et al. Biophys. J.2021, 120, 27663377396310.1016/j.bpj.2021.03.012PMC8390897]. We extend those findings by considering the effect of encapsulation on mRNA thermal unfolding with the observation that encapsulation in LNPs increases mRNA unfolding temperatures.

## Introduction

The spectacular advent of mRNA vaccines as a rapid response to the COVID-19 pandemic has ushered in a new front line in vaccinology. These vaccines combine the informational sophistication of the mRNA molecule with the physicochemical architecture of lipid nanoparticles to create a tunable drug delivery platform with great potential for broadly versatile medical applications.^2−5^ Understandably, the COVID-19 pandemic has focused much attention on the practical details of vaccine shelf life, storage conditions, supply, and distribution,^6^ while also highlighting that, well prior to this pandemic, poor vaccine thermostability^7,8^ coupled with supply chain logistics challenges^9^ had already been regarded as an Achilles heel of efforts to globally vaccinate against deadly infectious diseases. The goal of achieving more robust vaccine products connects directly to the understanding of the underlying molecular interactions and ultimately translating this knowledge into rational formulation design strategies.^10^ These connections have been well-developed for therapeutic proteins; however, for mRNA vaccines, structural details of the conformational state and physicochemical environment of the mRNA within the lipid nanoparticle (LNP) formulation are only beginning to emerge. The central drug delivery challenge of mRNA is more formidable than for proteins because the mRNA molecule must be delivered intracellularly whereas pharmaceutical proteins remain limited to druggable targets outside the cell. An addition to this challenge is the intrinsic rapid biodegradability of mRNA. Although this is a favorable attribute from a safety and pharmacokinetic perspective,^11^ unfortunately, it also confers susceptibility to degradation in vitro. A more detailed understanding of the pathways specifically relevant to mRNA pharmaceutical instability is currently emerging.^12^ Similarly, although lipid nanoparticles have been studied as drug carriers for about 30 years,^13^ the first approved product using this technology occurred only recently with ONPATTRO (patisiran) in 2018.^14^

To create perspective, it is intriguing to consider the current state of mRNA biomedical technology relative to therapeutic protein biotechnology and the advances made preceding and following FDA’s approval of the first recombinant DNA drug product, human insulin (Humulin), in 1982.^15^ This milestone was well preceded by the crystal structure of pig insulin in 1969^16^ with an elegant description of the underlying factors governing correct protein folding well-articulated by the early 1970s.^17^ A pathway to improved protein pharmaceuticals then unfolded (no pun intended), leveraging regulatory precedent and an understanding of the underlying structure and folding to create improved sequences conferring greater pharmaceutical stability and/or improved therapeutic properties.^18^ The approval of Humalog in 1996 provided a striking example of the latter, showing how even a subtle amino acid sequence modification could be engineered to dramatically alter insulin pharmacokinetics via a physicochemical mechanism,^19,20^ thereby addressing a therapeutic need. These biomedical milestones stand on the shoulders of historic advancements in recombinant DNA technology, protein engineering, and structural biology at atomic resolution serving to emphasize that the route to better biomedical products is based firmly on understanding the fundamental interrelationships among higher-order structure, biological activity, and biomedical implementation, aka the molecular pharmaceutics.

## The RNA Molecule

mRNA was discovered in 1961, and an indication of its biomedical potential was demonstrated in 1990 when protein production was shown from reporter gene mRNAs injected into mice.^21^ Historically, structural studies of RNA in the 1960s were part of a frenzy to understand their function in decoding genetic information. The ensuing discovery of catalytic RNA in the early 1980s was a major milestone in RNA science with the recognition that its 3-dimensional structure is intrinsically related to its function.^22,23^ Up until that time, X-ray crystallographic studies of RNA had focused predominantly on transfer-RNA (due in part to limitations of preparative methodologies for adequate quantities of other RNA types) and then ribosomal-RNA motivated by the fascination with its central role in protein biosynthesis.^24^ Early therapeutic proteins were more convenient to study. As relatively stable, naturally occurring molecules, they could be isolated from animal or human tissues in large quantities. For example, insulin could be purified in gram quantities and had been used therapeutically since the 1920s. Large-scale global production was achieved within a few years of its discovery, and commendably, advanced pharmaceutical formulations had been developed by the 1930s.^25^ The biomedical potential of insulin and the full knowledge of its 3-dimensional structure had thus been thoroughly established well before its launch as the first biosynthetic human protein drug in 1982. In fact, structurally, biochemically, medically, and pharmaceutically, insulin was one of the most thoroughly studied protein molecules in history by that time.

In contrast to proteins, the concept of mRNA as a drug or vaccine is relatively recent, one that faced formidable drug delivery challenges and was not preceded by a long history of applicable pharmaceutical sciences research. A 3-dimensional structure of our mRNA molecule is not available, and an understanding of the interrelationships among higher-order structure, protein translational efficiency, and chemical stability is only beginning to emerge.^26−30^ Indeed, it is amusing to note that as late as the mid 1990s RNA scientists practically lamented the reverence that protein scientists held toward the delicacy of higher-order structure,^31^ apparently reflecting the more contemporary appreciation of RNA folding. mRNAs are large molecules (typically ≈1000–4000 nucleotides): three nucleotide units code for each amino acid of the protein for which they encode, and each nucleotide has a mass of about 330 Da versus the average amino acid mass of 110 Da. The incorporation of ribose sugars into RNA as opposed to deoxyribose sugars for DNA makes RNA more suitable for its short-lived purpose in vivo,^32^ whereas DNA (the information storage molecule) is highly stable to the extent of boasting an impressive 521-year half-life.^33^ Chemical modifications to the mRNA molecule including cap structures and modified nucleosides have played a key role in overcoming challenges of immunogenicity, achieving prolonged stability and potent, accurate protein expression in vivo.^5,34^ These advancements have been central to enabling exogenous mRNA delivery; however, the achievement of the ideal target product profiles for mRNA vaccines and therapeutics will require further innovative approaches.

**Figure 1:**
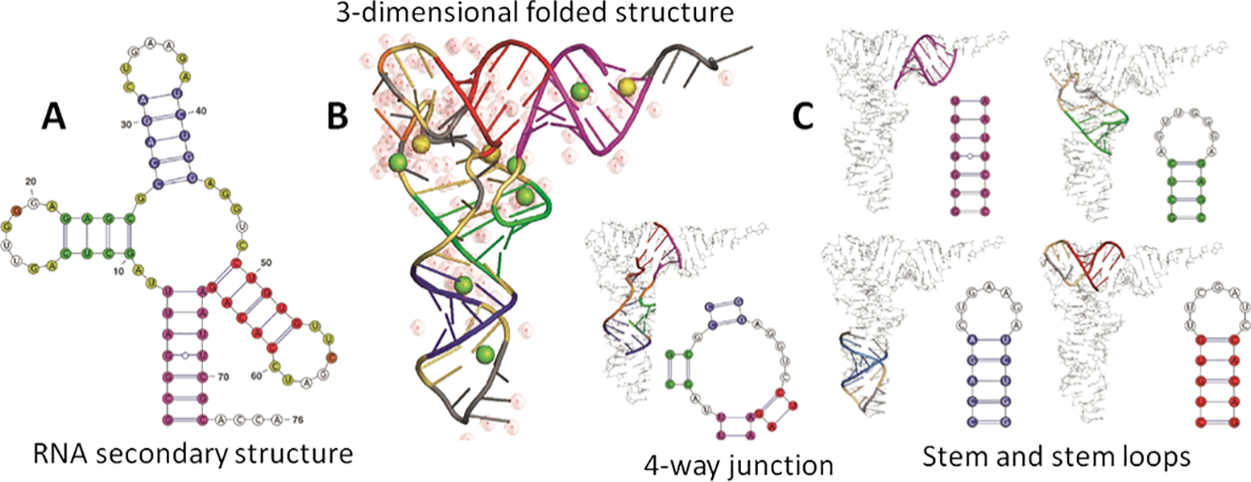
Representations of RNA higher-order structure showing how secondary structures formed from complementary base pairing fold into 3-dimensional tertiary structures. (A) RNA secondary structure; (B) 3-dimensional tertiary structure showing water and ions; (C) the incorporation of specific secondary structural elements into the tertiary structure. Figure adapted from Dawson, W. K.; Bujnicki, J. M. Computational modeling of RNA 3D structures and interactions. *Curr. Opin. Struct. Biol.***2016***37*, 22 (ref (90)) under the terms of creative Commons license http://creativecommons.org/licenses/by/4.0/.

mRNA is a single stranded molecule that can form double stranded structures by folding over on itself, forming hairpin stem-loops and pseudoknots stabilized by the intramolecular hydrogen bonds formed through complementary pairing of contiguous bases. These secondary structures fold into 3-dimensional tertiary structures as illustrated in Figure 1.

RNA is a polyanion with complex conformational and charge interaction behaviors in solution. The negative charges on the chain repel each other causing the RNA molecules to stretch out in solution. However, this stretching lowers the conformational entropy so that the molecule arrives at an optimum between minimizing the repulsive potential of like charges by stretching out as much as possible while keeping the entropy sufficiently positive. In the presence of an electrolyte, the charges will be shielded, and the molecule will relax electrostatically and shrink in size. Consequently, the folding of RNA into stable tertiary structures is extremely sensitive to counterions^35^ or, in the parlance of the pharmaceutical scientist, expected to be highly formulation dependent. Although Ca^2+^ and Mg^2+^ play important roles in the stabilization of RNA folding in vivo by participating in the ionic microenvironment as diffuse ions or directly bound at high affinity sites, divalent cations also promote cleavage of RNA.^36,37^ Large RNA molecules in solution are generally considered to be more extended and diffuse than globular proteins but still adopt diversely complex sequence-dependent higher-order structures.^38^ While much progress has been made in the prediction of the secondary structure from the sequence, the proverbial “rugged energy landscape” makes the prediction of the tertiary structure a formidable problem, especially for the very large mRNA molecule.^39^ Both proteins and RNA exhibit secondary and tertiary structures dependent on the primary sequence; however, protein higher-order structures are driven primarily by the burial of the hydrophobic side chains whereas RNA folding is a hierarchical process driven predominantly by base stacking, hydrogen bonding, and electrostatic stabilization.^40^ Although there is a superficial parallel between protein- and RNA-higher-order structures, the analogy breaks down upon deeper consideration of structure–function interrelationships. For mRNA specifically, Crick’s classic metaphor of mRNA as an audio tape running through the reading head of a tape recorder emphasizes the significance of the primary sequence as the central basis of its function, carrying the genetic code from DNA to ribosomes that unwind the folded mRNA molecule to decode the message. In contrast, it was the 3-dimensional folded structure of proteins that historically held such fascination and is most associated with launching the field of structural biology. It follows that the preservation of the integrity of a therapeutic protein’s folded structure has always been synonymous with achieving good pharmaceutical stability, whereas fundamental details of how mRNA folding relates to its biological activity are still emerging.^30^

The comparison of the crystallization behavior highlights the physicochemical differences. The crystallization process is a form of molecular recognition, making its manifestation a visible outcome of the underlying structural and interfacial phenomena. In a comparison of proteins with RNA molecules, the differing chemical nature of the repeating units in these two types of biopolymers results in quite different propensities for molecular ordering and crystallization. Many pharmaceutical proteins crystallize well because the molecular characteristics and solution conditions that favor crystallization also favor pharmaceutical integrity and stability (purity, chemical homogeneity, conformational homogeneity, and solubility). Indeed, their crystallization conditions can provide useful clues for the identification of optimal pharmaceutical formulation conditions because the crystallization process is sensitive to multiple pharmaceutical quality attributes.^41^ In contrast, large RNA molecules are highly dynamic and are characterized by extensive conformational heterogeneity with poorly differentiated molecular surfaces that offer few tertiary contacts to favor lattice formation. The very large size of mRNA molecules further compounds the difficulty of achieving a well-ordered crystal lattice, as evident from the description by Gopal et al. of the long RNA molecule in solution as “a statistical object whose properties are derived from an ensemble of possible structures”.^42^ Although many RNA structures have been reported in the structural biology literature and open databases, these predominantly correspond to comparatively short sequences, tRNA, and/or RNA in complexes with proteins.^43^ In contrast, many therapeutic proteins, including monoclonal antibodies, have X-ray crystal structures defining their 3-dimensional structures. Prospects for mRNA structures with atomic level precision are poor because, in practice, NMR and X-ray crystallographic methods are restricted to relatively small sequences (<≈200 nucleotides).^44^

## Lipid Nanoparticles for mRNA Delivery

Due to their size, polyanionic nature, and hydrophilicity, the intracellular delivery of mRNA molecules cannot generally rely on passive diffusion across cell membranes. Furthermore, nucleic acids are rapidly degraded by endogenous nucleases in physiological fluids. To overcome these challenges, LNPs incorporating ionizable lipids have been developed as delivery vehicles for siRNA and mRNA, serving both to protect the delicate cargo from degradation in vivo and to enable delivery into the cell. LNPs for mRNA delivery generally comprise a zwitterionic phospholipid, cholesterol, a polyethylene glycol (PEG) lipid, and an ionizable lipid.^45,46^ Engineering the lipid structure^47^ and particle surface has thus been pursued as a strategy to enhance cellular uptake and endosomal escape^48,49^ while specific nanostructural features of the LNP, such as nonlamellar lipid phases, have also been suggested to affect delivery efficiency.^50^ LNP interfacial interactions with the cell membrane initiate the internalization of the mRNA; then, inside the cell, the more acidic environment protonates the ionizable lipid, reversing the electrostatic interactions to release the RNA cargo for translation and subsequent protein expression. This is of course an oversimplified description, and the reader is directed to some excellent recent reviews that address these intracellular aspects in more detail.^45,51^ The LNP morphology, lipid packing, and microenvironment of the encapsulated mRNA are thus plausibly connected to the delivery efficiency, emphasizing the central significance of higher-order structure associated with both the mRNA and LNP.

The challenges of LNP preparative scale-up and large-scale processing represent another interesting point of distinction between mRNA-vaccines and biologic drugs. While LNPs may be prepared at bench-scale using pipets and simple manual mixing,^52^ their cGMP scale-up for clinical/commercial use employs finely controllable rapid-mixing platforms involving multiple processing steps.^53,54^ In contrast, the large-scale production of many biologic drugs can be based on relatively simple thaw–filter–fill drug product production processes. On the other hand, the operational simplicity of the drug product is offset by the complexity of biologic drug substance production processes, which are based on microbial fermentation or mammalian cell culture. mRNA is produced synthetically at large scale using the in vitro transcriptase reaction.^55^

**Figure 2:**
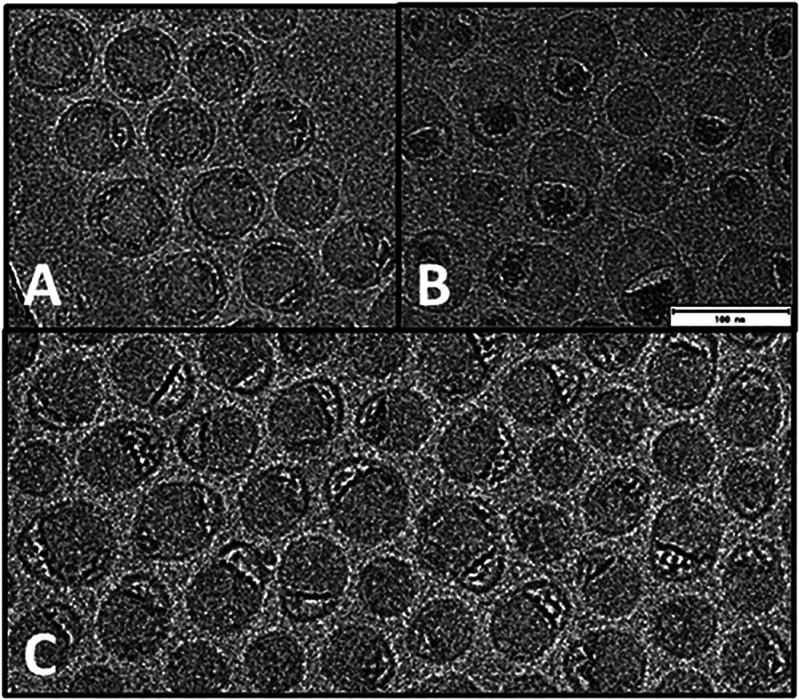
Cryo-EM characterization of the representative mRNA-LNP preparations. When the mRNA was highlighted with a permeating cationic dye, it became evident that LNPs may form morphological arrangements corresponding to different states of mRNA-lipid association. Panels A and B show extremes of highly spherical versus nonspherical morphologies where, in the latter, mRNA is dissociated from the solid lipid body of the nanoparticle to reside in a bleb compartment. Panel C shows the significant degree of nanoheterogeneity that can be present including states of mRNA-lipid association intermediate between panels A and B. Figure adapted from Brader et al. Encapsulation state of messenger RNA inside lipid nanoparticles. *Biophys. J.***2021**, *120* (14), 2766–2770 (ref (1)) under the terms of Creative Commons license http://creativecommons.org/licenses/by/4.0/.

**Figure 3:**
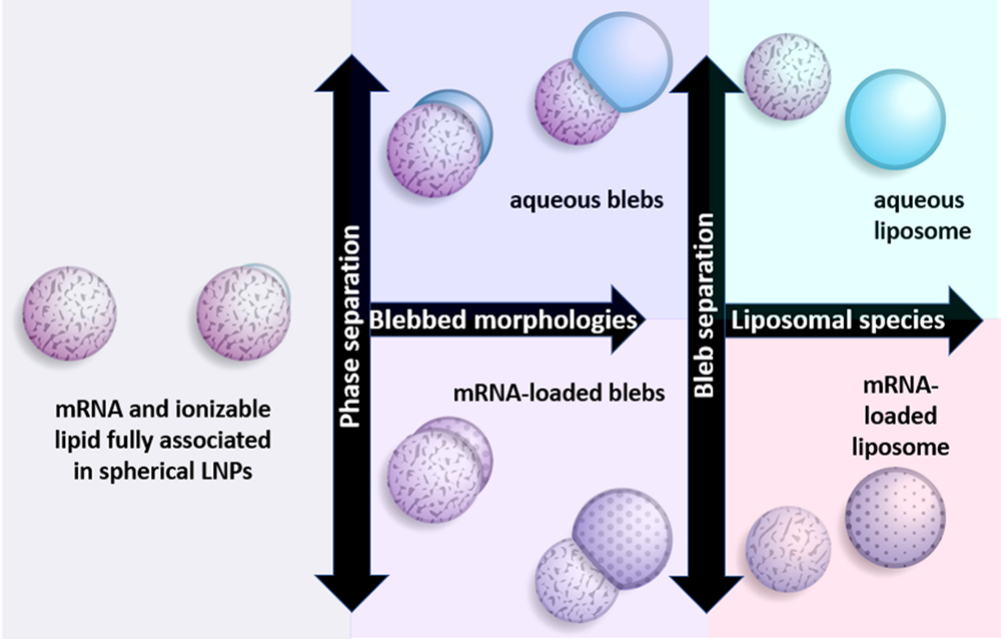
Schematic representation of mRNA-LNP morphologies. At the far left, a spherical particle with mRNA and the lipid fully associated is depicted. The formation of a bleb involves the creation of a protrusion on the LNP characterized by a darkened, thicker periphery consistent with enrichment of the DSPC as the most mass dense component present. This aqueous compartment can occur to varying degrees and may or may not be occupied by mRNA (blue depicts aqueous; mottled surface depicts mRNA). In the extreme of dissociation, at right, mRNA-loaded or unloaded liposomes may also be formed as minor components.

Although cryo-EM images of mRNA-LNPs reported in the literature often appear as relatively featureless gray circles, we were intrigued by the diversity of mRNA-LNP morphologies in some published images where spherical and nonspherical morphologies are apparent, including blebs (cavernous protrusions) of varying size, sometimes showing resolved striations indicative of annular arrangements.^56−58^ The literature indicates there are many processing and compositional factors that can influence the outcome of the nanoprecipitation reaction, and it is also apparent that the chemical nature of the component lipids can affect the LNP morphology,^48^ even to the extent of producing distinctive faceted particle geometries.^50^ The siRNA-LNP literature reflects a fairly concordant description of the LNP assembly supported by biophysical studies. Presumably, due to their small size (≈20 nucleotides) and double stranded character, the siRNA molecules can be more readily accommodated within a structural arrangement directed predominantly by preferences of the lipids. The much larger mRNA molecules evidently require a different accommodation. A direct comparison of LNP formation using siRNA versus mRNA under the same LNP preparative scheme showed that mRNA formed LNPs with very large bleb features, whereas the siRNA-LNP counterpart were devoid of these.^56^ When one recognizes that cryo-EM mass density in the blebs can be assigned to mRNA, it follows that the nature of the encapsulated RNA molecule itself can influence LNP morphology and the degree of lipid association. Clearly, the encapsulation of large mRNA molecules is more complex than common representations of the LNP as a sphere with RNA lipid-bound in the core.^59^ On the basis of our recent study pinpointing the location of mRNA within the LNP,^1^ we suggest that LNPs may be better represented as a continuum of states corresponding to varying degrees of mRNA-lipid association as shown in Figures 2 and 3.

## Conformational Stability and Pharmaceutical Stability

The lipidic environment of encapsulated mRNA involves several chemical components, creating the possibility that each pharmaceutical raw material may adversely affect the mRNA stability profile.^12^ Structural effects of the encapsulation itself on the mRNA 3-dimensional fold may also conceivably influence stability by exposing or burying labile regions. The mRNA molecule within the LNP is thus expected to possess a distinct stability profile relative to naked RNA. It is worth emphasizing at this point that the term “stability” is often used in different scientific contexts across the pharmaceutical sciences and biological sciences fields. It has been described basically as the potential of a pattern to survive over time,^60^ and in the context of a biological-based medicinal product, the relevant pattern may be categorized broadly as either conformational or compositional. Denaturation, unfolding, self-association, and aggregation relate to the former, whereas processes creating new chemical entities such as cleavage, enzymatic degradation, oxidation, and covalent adducts^12^ are examples of the latter. The preservation of correct higher-order structures, specifically secondary, tertiary, and quaternary structures, represents a central focus of biologic product development and characterization^61^ for which the close connection between conformational stability and chemical stability is well recognized.^62^ Interfacial stresses associated with large-scale production, filling, shipping, storage, handling, and administration create formidable challenges to preserving these delicate higher-order structures necessary for biological activity.^63^ Signatures of thermal unfolding can thus provide some basis for understanding how pharmaceutical conditions impact the intrinsic structural stability with a view to identify solution conditions robust to large-scale processing and pharmaceutical stresses while also enabling a convenient comparison of sequence variants in discovery phases of drug development.^64^ A prediction of the shelf life of proteins from accelerated data remains largely aspirational, nevertheless, gaining predictive insights into the overall pharmaceutical stability of formulated drug products by screening thermal unfolding signatures featured prominently in protein developability workflows within the biotechnology industry.^65−67^ Similarly, there is evidence that the secondary structure affects the chemical stability of RNA.^68^ We were interested in exploring the impact of encapsulation on the thermal folded stability of the mRNA molecule with a view to understand its potential relevance to pharmaceutical stability.

## Thermal Unfolding

**Figure 4:**
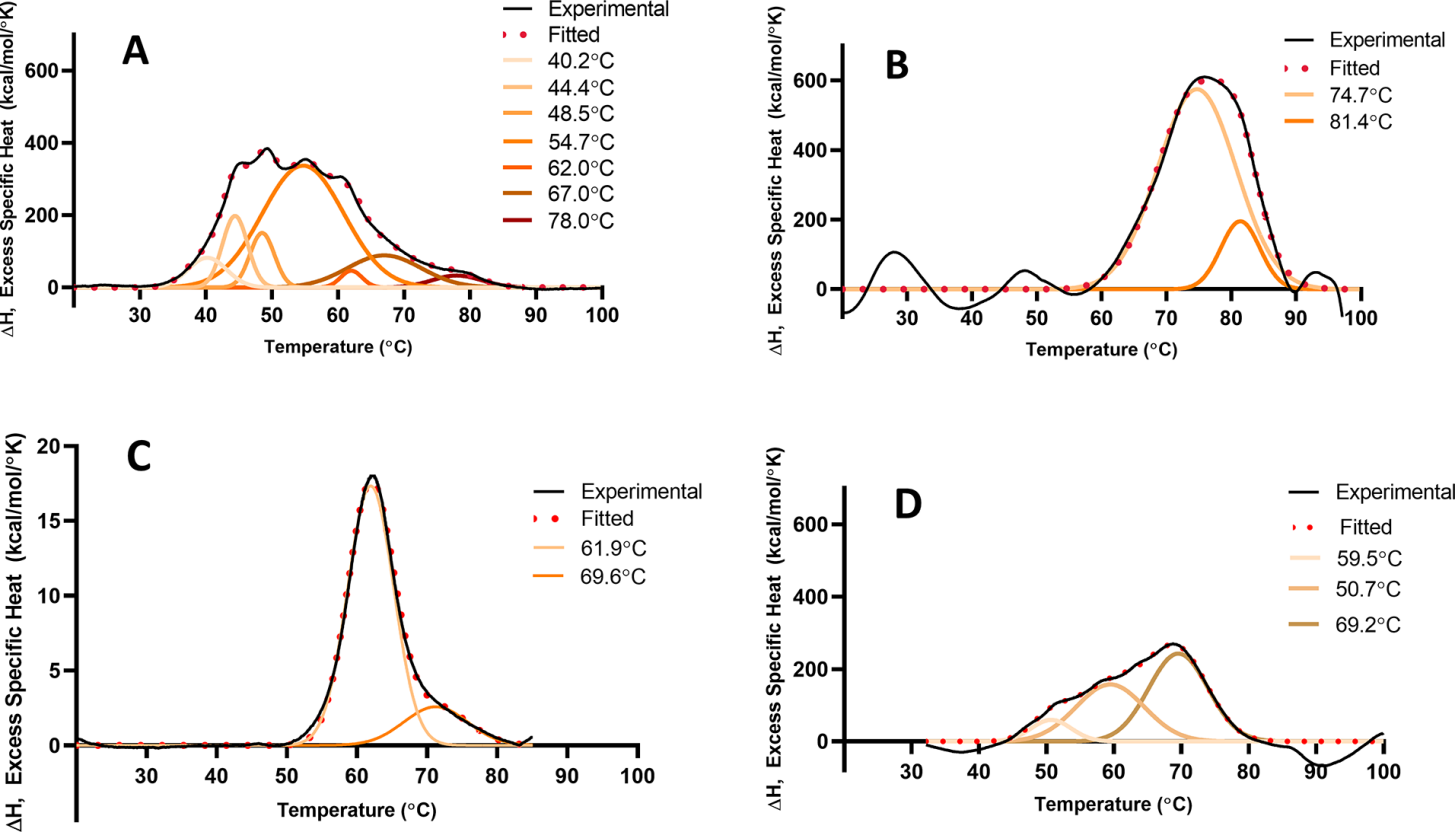
Differential scanning calorimetry thermograms: (A) an unencapsulated mRNA; (B) same mRNA encapsulated in LNP, both in 20 mM Tris, 8% sucrose, and pH 7.4 buffer; (C) bovine serum albumin in 5 mM sodium citrate, pH 6.5 buffer; (D) mRNA-LNP sample from (B) reheated (after it had been heated to 95 °C and then cooled back to room temperature). In each panel, the experimental data is shown as the black line together with the mathematical fit in red. Gaussian components of the fit are shown as a color gradient together with the midpoint temperatures of each transition. Data were recorded using a MicroCal PEAQ-DSC capillary differential scanning microcalorimeter (Malvern-Panalytical, Malvern, United Kingdom) with a scanning rate of 1.5 °C/min.

Thermal melting techniques for RNA are well-developed and have been applied extensively to studying nucleic acid folding and stability.^69^ These approaches utilize optical or calorimetric readouts to detect conformational change as the molecule unfolds. Much historical impetus for this work has come from the goal of understanding biologically relevant conformational transitions, as these occur via breakage and formation of base pairing interactions. The thermal stability of secondary structural elements in mRNA has received additional attention recently due to emerging evidence that this attribute relates to actual function (protein expression).^27,70^ We applied differential scanning calorimetry (DSC)^66^ to a free mRNA to produce the thermogram shown in Figure 4A.

A complex thermogram requiring multiple Gaussian components to produce an adequate fit indicative of a complex thermal unfolding pathway is evident. As an illustrative comparator, we also recorded the DSC of bovine serum albumin (BSA), a “model globular protein” (Figure 4C). BSA exhibits a much simpler thermal profile even though its 3-dimensional structure comprises 3 domains containing significant proportions of an α-helix, β-sheet, and random coil.^71^Figure 4B shows the DSC signature of the same mRNA formulated as LNPs, revealing that significant changes in the thermal profile occurred together with an overall shift of the mRNA signal to higher temperatures. The data of Figure 4A,B show that, unencapsulated, the mRNA thermogram required 7 Gaussian fitting components, whereas encapsulated, the profile could be fit with only 2. When the mRNA-LNP sample corresponding to Figure 4B was rescanned after cooling back down to room temperature, the thermogram shown in Figure 4D was obtained, which is different from both free mRNA and mRNA in LNP, exhibiting transitions that have shifted to lower temperature relative to mRNA in LNP. This again suggests a protective effect of encapsulation within the LNP that is abolished when the LNP structure is denatured by heat. DSC unfolding of free mRNA is characterized by the existence of many closely spaced and rather sharp transitions, probably corresponding to many similar, but different size, structural elements. When encapsulated in the LNP, those structural elements are transformed into a smaller number of more stable “superstructures”.

**Figure 5:**
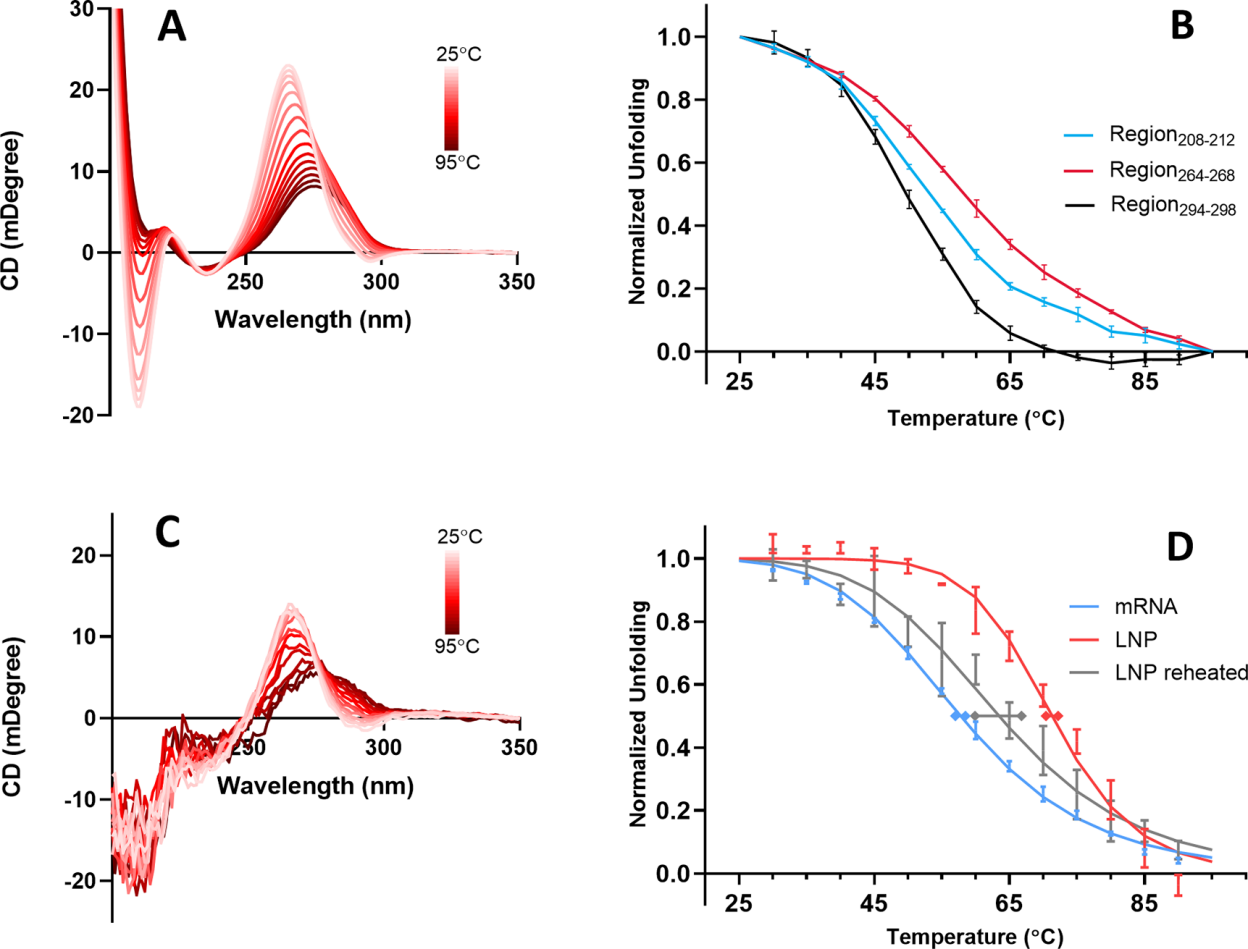
(A) Circular dichroism spectra of mRNA recorded on a Jasco J1500 spectrometer at successively increasing temperatures of 5 °C increments from 25 to 95 °C. (B) Thermal unfolding trajectories measured from integrated areas of the three wavelength regions indicated, which correspond to distinct CD spectral bands. (C) CD spectra of mRNA-LNP recorded at successively increasing temperatures in 5 °C increments from 25 to 95 °C. (D) Thermal unfolding of mRNA measured by monitoring the CD spectral area in the region of 264–268 nm. Shown in blue is the unfolding curve of free mRNA (in buffer). The red data corresponds to the unfolding trajectory of the same mRNA encapsulated in LNP. The gray curve corresponds to the same mRNA-LNP sample cooled from 95 °C and then heated again from 25 °C. Experimental averages and standard deviations are based on 3 independent experiments. Normalized data, *y*, were fit with the Hill equation, *y* = 1/[1 + (*T*_m_/*T*)^Hill^] where the superscript “Hill” refers to the Hill coefficient. *T*_m_ and Hill coefficient values are presented in Table 1.

To further evaluate the DSC results using a different biophysical technique, we applied circular dichroism (CD) spectroscopy, which can be applied to the characterization of the conformation of nucleic acids in solution.^72^ CD spectra of nucleic acids are mostly dependent on the sequence and stacking geometry of the bases, and in the case of short RNA sequences, it is possible to distinguish A-form, B-form, and Z-form helices.^73^ The CD spectrum of mRNA at 25 °C, shown in Figure 5A, is qualitatively indicative of the A-form helix with two negative bands (210 and 295 nm) and one positive band (265 nm). This profile is similar to spectra reported previously for mRNA and tRNA.^74,75^ CD spectra of this mRNA recorded at 5 °C temperature increments from 25 to 95 °C are also shown in Figure 5A. It is apparent that the spectrum changes in a complex way with different wavelength regions shifting relative to one another, a tell-tale sign that the effect of heating is not simply to produce a change in the proportions of two states (folded and unfolded). Instead, the result suggests a complex mix involving multiple conformations. This interpretation is also apparent from monitoring the CD change at a series of specific wavelength regions corresponding to the CD bands, each of which resulted in distinctly different unfolding trajectories (Figure 5B). We infer that different regions of the mRNA molecule are affected by heat in unique ways, and as a result, the unfolding of these regions occurs along different pathways. This type of interpretation has been postulated previously from CD melt experiments on RNA.^76^

The effect of encapsulation on the thermal melting of mRNA was also characterized using the CD signal. The CD spectrum of encapsulated mRNA recorded at 5 °C increments is shown in Figure 5C. From these spectra, the ellipticity of the 264–268 nm region was monitored upon heating (thereby avoiding interference from cholesterol CD, which coincides with the 208–212 nm band). The comparison of encapsulated versus free mRNA showed a similar effect to the DSC results (Figure 4) with the thermal unfolding profile shifted to significantly higher temperatures as a result of encapsulation. Intriguingly, when the heated mRNA-LNP solution was cooled to room temperature and then reheated, the resulting mRNA profile had shifted significantly to become much more comparable to the melt of the unencapsulated mRNA. This result suggests that it is the specific nanostructured encapsulation state (which was destroyed upon heating to 95 °C) that enhances mRNA folded stability.

For proteins, the assignment of CD bands to specific secondary structural elements has been well-established on the basis of the far-UV CD of the peptide bond.^77^ The determination of the protein structure from the CD spectra is possible because the far-UV CD arises from the intrinsic CD of the protein backbone. In contrast, much of the nucleic acid CD originates from the stacking geometry and sequence-dependent base pair content, making the relationship between the nucleic acid secondary structure and CD spectral shape more convoluted than is the case for proteins, especially in the case of a large single-stranded mRNA. With this caveat, we offer some interpretations of the spectral features present in Figure 5A. Published experimental results indicate that the strong negative band observed at 210 nm is the result of nearest-neighbor interactions and base stacking.^78,79^ Studies on DNA have found that a particularly large spectral effect originates from guanine–guanine stacking, which makes an important contribution to the formation of helix duplexes, leading to the strong positive band at 265 nm.^80^ In general, a positive band in the 260–280 nm region suggests the formation of a right-handed helix.^72^ A dominant positive band at 260 nm and a sharp negative band at 210 nm characterize the CD spectra of A-form helix RNA.^81^ The weak band observed at 295 nm has been identified as the result of the nearest-neighbor interactions.^78^ In thermal melting experiments, CD changes are primarily caused by base destacking, leading to the loss of interaction between neighboring bases, which manifests as a decreased magnitude of CD bands. In the free mRNA spectrum of Figure 5A, the most thermally susceptible region appears to be the 295 nm band followed by the 210 nm band. A simplistic interpretation, the unstacking of the single weakest neighboring bases, is the first event observed during mRNA heating (295 nm), leading to further unzipping of longer sequences observed as melting at 210 nm. These events lead into a major helix to coil transition, affecting the whole double helical structure evident at 265 nm. The Hill coefficient has been calculated from sigmoidal CD melting curves as an indication of cooperativity of RNA global unfolding.^82^ The Hill coefficient values obtained from the curves of Figure 5D are given in Table 1. These values indicate that cooperativity of mRNA unfolding within the LNP is significantly higher than that of mRNA in solution or in the reheated LNP. We can infer that mRNA encapsulated in the LNP is structured differently and more compact than mRNA in solution or in heat denatured LNP.

**Table 1:** Transition Midpoints (*T*_m_) and Hill Coefficients (Hill) Obtained from the CD Data at 3 Spectral Bands Shown in Figure 5

	CD spectral band					
	208–212 nm		264–268 nm		294–298 nm	
sample	*T*_m_ (°C)	Hill	*T*_m_ (°C)	Hill	*T*_m_ (°C)	Hill
free mRNA	53.0 ± 0.2	–6.3 ± 0.4	58.4 ± 0.8	–6.2 ± 0.1	49.6 ± 0.5	–9.2 ± 0.3
mRNA in LNP			71 ± 1	–11 ± 1	60.7 ± 0.4	–5 ± 1
mRNA in LNP reheated			64 ± 3	–6.7 ± 0.7	63 ± 4	–11 ± 6

A comparison of events occurring in free versus encapsulated mRNA leads to the conclusion that mRNA becomes thermally more stable in its encapsulated form, more effectively preserving its basic CD signature during heating. This observation parallels a pharmaceutical stabilization strategy used for proteins whereby enhanced folded stability may be conferred by favorable formulation conditions.^67^ Here, we show that some degree of analogy exists regarding LNP encapsulation conferring enhanced conformational stability to the mRNA molecule. The achievement of the overall pharmaceutical stability of lipid nanoparticles will also require consideration of colloidal stability. This raises some further interesting analogies and contrasts to proteins as net attractive or repulsive forces between molecules or particles can influence pharmaceutical stability. With the advent of monoclonal antibodies (mAbs) as a prominent class of therapeutics and the frequent need to deliver them at high dose and high concentration, favorable colloidal properties have become increasingly critical to achieve overall pharmaceutical stability and deliverability.^83^ It is not uncommon to formulate mAbs at >100 mg/mL under which condition the distance between van der Waals surfaces of the molecules is on the same order of magnitude as the size of the molecules themselves.^84^ Consequently, solubility, stability, and viscosity become strongly influenced by intermolecular networks that form in solution.^85^ These networks have a complicated dependence on the subtleties of surface morphology, dipole character, and patchiness of the electrostatic and hydrophobic surfaces. Although monoclonal antibodies are large molecules with hydrodynamic diameters in the vicinity of ≈5–7 nm, this size is considerably smaller than the ≈100 nm size of the lipid nanoparticles used to deliver RNA. Consequently, colloidal aspects can be expected to play a key role in their formulation. While LNPs may be cartoonishly represented as uniform spheres, the images in Figure 2 show potential for significant complexity of size, shape, surface composition, surface electrostatics, solvation, and proximity energies.^1,86^

## Conclusions

Early descriptions of mRNA emphasized its instability. Titles of the landmark publications announcing its discovery referred to the molecule as “An unstable intermediate...”^87^ and “Unstable ribonucleic acid...”.^88^ While these descriptions were certainly not made with pharmaceutical applications in mind, they were an indicator of the unique stability profile relative to other biopolymers. Because mRNA science has not evolved with a central emphasis on biomedical applications, the interrelationships among primary sequence, higher-order structure, biological activity (protein expression), and pharmaceutical stability have only relatively recently become the focus of intense research. The accelerated thermal degradation of proteins has been studied extensively throughout biopharmaceutical history^89^ as a means of connecting fundamental thermodynamic measurables to the ultimate practical resilience of the intended therapeutic product. While there is clearly a unique set of challenges associated with stabilizing mRNA vaccines, the observation that encapsulation within LNPs significantly enhances the folded stability of mRNA strikes a familiar and optimistic note.
